# Optimizing Autonomic Function Analysis via Heart Rate Variability Associated With Motor Activity of the Human Colon

**DOI:** 10.3389/fphys.2021.619722

**Published:** 2021-06-29

**Authors:** M. Khawar Ali, Lijun Liu, Ji-Hong Chen, Jan D. Huizinga

**Affiliations:** ^1^Faculty of Engineering, School of Biomedical Engineering, McMaster University, Hamilton, ON, Canada; ^2^Division of Gastroenterology, Department of Medicine, Faculty of Health Sciences, Farncombe Family Digestive Health Research Institute, McMaster University, Hamilton, ON, Canada

**Keywords:** high-amplitude propagating pressure waves, RMSSD, RSA, Baevsky’s Stress Index, autonomic nervous system, colonic motility

## Abstract

The parameters of heart rate variability (HRV) can non-invasively assess some autonomic activities, and HRV is influenced by many bodily actions. Although parasympathetic activity is the primary driver of colonic propulsive activity, and sympathetic activity a major inhibitor of colonic motility, they are rarely measured and almost play no role in diagnosis of colon motor dysfunction or in standard treatments. Here we set out to optimize HRV analysis of autonomic nervous system changes related to human colon motility. The electrocardiogram and impedance were recorded in synchrony with colonic motor patterns by high-resolution manometry. Respiratory sinus arrhythmia (RSA), root mean square of successive differences of beat-to-beat intervals (RMSSD), the Baevsky Index or Sympathetic Index (SI), and the ratios of SI/RSA and SI/RMSSD were shown to indicate a marked increase in parasympathetic and withdrawal of sympathetic activity during the high-amplitude propagating pressure waves (HAPWs). Strong associations were seen with HAPWs evoked by a meal and rectal bisacodyl indicating a marked increase in parasympathetic and withdrawal of sympathetic activity during the gastrocolic reflex and the defecation reflex. When HAPWs occurred in quick succession, parasympathetic activation (RSA and RMSSD) occurred in a rhythmic fashion. Hence, during propulsive motor patterns, an overall shift in autonomic activity toward increased parasympathetic control was shown to be reflected in HRV. HRV assessment may therefore be valuable in the assessment of autonomic dysfunction related to colonic dysmotility.

## Introduction

Measurement of autonomic function does not yet play a significant role in colon dysmotility diagnosis, despite the fact that propulsive contractions in the human colon are orchestrated by the parasympathetic nervous system (De Groat and Krier, 1976; Browning and Travagli, 2019). Some studies have linked gastrointestinal activity such as the postprandial state (Lu et al., 1999) and gastric hypersensitivity (Ouyang et al., 2020), to high frequency (HF) and low frequency (LF) parameters. Autonomic activity associated with Irritable Bowel Syndrome (IBS) (Bharucha et al., 1993) and chronic intestinal pseudo obstruction (Camilleri et al., 1993) were studied using heart rate interval parameters, heart rate response to deep breathing and other tests. Autonomic function associated with functional dyspepsia was studied using HF power and root mean square of successive differences (RMSSD) (Lorena et al., 2002).

Heart rate can react momentarily to changes in nervous input from the autonomic nervous system to the sinoatrial node, and this property establishes heart rate variability (HRV) (Thayer et al., 2012; Baevsky and Chernikova, 2017; Shaffer and Ginsberg, 2017). Several time and frequency domain analyses and non-linear methods have been developed to analyse HRV. Especially spectral analysis of beat-to-beat intervals, assessing the band power of low-frequency (LF; 0.04–0.15 Hz) and high-frequency (HF; 0.15–0.4 Hz), are used as matrices of the sympathetic and parasympathetic nervous systems (Hayano and Yuda, 2019). The HF band power is considered a measure of parasympathetic nervous system activity, the effect of activity of the final vagal fibers innervating the sinoatrial node, a culmination of vagal innervation that was influenced by a myriad of factors, primarily breathing but also activity from regulatory nuclei such as the nucleus tractus solitarius (NTS) that orchestrate coordination between respiratory, cardiac and gastrointestinal activities to optimize responses to metabolic demands and hence influence the autonomic outflow to the heart (Grossman and Taylor, 2007; Browning and Travagli, 2014; Singh and Jaryal, 2020). Dendritic projections from efferent vagal motor neurons to the colon extent throughout the NTS and intermingle within the various subnuclei so as to co-ordinate homeostatic reflexes across autonomically controlled organs (Browning and Travagli, 2014). The NTS is a key structure for autonomic and neuroendocrine integration (Jean, 1991). The coordination of gastrointestinal, respiratory and cardiac function is dramatically seen in cases of emergency. The afferent information from the airways is first processed at the level of NTS and results in various reflexes that are required for modification of ongoing breathing along with modulation of autonomic output to the cardiovascular and respiratory systems (Singh and Jaryal, 2020). It may be assumed that similar processes are involved in the central control of colon motility where the NTS plays a critical role (Browning and Travagli, 2014).

Effects of organ activities on HRV are difficult to predict and sometimes counterintuitive (La Rovere et al., 2003). The major motor pattern of the human colon, the high-amplitude propagating pressure wave is associated with the autonomic nervous system in two ways. It is orchestrated by the parasympathetic and enteric nervous system and its occurrence, due to increased intraluminal pressure and distention of the colon, will activate stretch sensitive neurons. The role of the autonomic nervous in orchestrating colonic motility is exemplified by the sacral defection reflex (Bharucha and Brookes, 2018) that starts with rectal sensation, which activates the sacral sensory nerves. Then information is signaled into the sacral parasympathetic nucleus (also called the sacral defecation center) from where information travels into the brain stem and frontal cortex to either prevent or initiate a defecation. A bowel movement may be produced via activation of sacral parasympathetic nerves and via the enteric nervous system (Browning and Travagli, 2014; Furness et al., 2014; Brookes et al., 2016; Bharucha and Brookes, 2018). The primary driver of the HAPWs is the parasympathetic nervous system (Devroede and Lamarche, 1974; De Groat and Krier, 1978; Callaghan et al., 2018). Resection of the parasympathetic pelvic splanchnic nerves causes loss of the defecation reflex (Devroede and Lamarche, 1974). HAPWs are not observed in ex vivo preparations of the human colon (Dinning et al., 2016). In the cat, HAPWs and HAPW-SPWs were identified in vivo and shown to be associated with firing of parasympathetic efferents (De Groat and Krier, 1978). Stimulation of sacral extrinsic nerves has also been shown to be a treatment for constipation (Leblanc et al., 2015). Interestingly, propulsive motor patterns can be evoked by injection of a ghrelin agonist in the sacral spinal cord (Shimizu et al., 2006) or by stimulation of surgically placed electrodes in the S2 region of the spinal cord (Devroede et al., 2012).

This study was designed to evaluate which of the myriad of HRV parameters best reflect autonomic nervous system activity using an established supine to standing protocol, and autonomic tone and reactivity associated with the high-amplitude pressure wave that is associated with human colon transit and defecation. We included HF and LF power, to directly compare RSA and HF power for statistical analysis, and to compare the disputed LF power as a measure of sympathetic activity with the Baevsky Index. We also separated the analysis by intervention, so that we could assess shifts in autonomic activity during HAPWs in response to a meal (the gastrocolonic reflex), and in response to rectal bisacodyl (the sacral autonomic (defecation) reflex, and in response to distention. We chose a combination of high amplitude propagating pressure waves (HAPWs) and high amplitude propagating pressure waves followed by simultaneous pressure waves (HAPW-SPWs) to incorporate all individual HAPWs in the statistical analysis, *excluding* in this analysis bisacodyl-induced multiple HAPWs since they sometimes are accompanied by pain and changes in breathing pattern. For bisacodyl-induced multiple HAPW activity we devised a new method for continuous assessment of HRV parameters. To study shifts in autonomic balance we propose new ratios of sympathetic over parasympathetic parameters.

## Materials and Methods

### Participants

Eleven healthy volunteers (7 males, 4 females, age 30 ± 10 years) without any current or prior history of cardiovascular or gastrointestinal disease and not on any medications affecting cardiac or gastrointestinal function were recruited by local advertisement (wall posters) for this study. Each participant was paid 600 CA$ to complete this study. The study was carried out at McMaster University with ethics approval from the Hamilton Integrated Research Ethics Board, and written consent from all participants.

### Heart Rate and Impedance Measurements

The electrocardiogram (ECG) was recorded using seven electrodes on the subject’s torso. Three electrodes formed a modified Lead II configuration for ECG recording. Four electrodes were used in a standard tetrapolar electrode configuration for impedance recording, where two electrodes supplied a constant current source, and two electrodes registered the changes in the transfer impedance (reflecting changes in activity of the sympathetic nervous system). ECG and impedance were recorded using a MindWare impedance cardio GSC monitor with a sampling frequency of 500 Hz. (MindWare Technologies Ltd., Gahanna, OH, United States) and MindWare BioLab Recording Software. MindWare HRV 3.1 was used for artifact correction of the ECG signal, to generate beat-to-beat intervals (RR intervals) and for the calculation of RSA, RMSSD, HF and LF band powers. PEP was generated by Mindware Cardiac Impedance software (MindWare Technologies Ltd., Gahanna, OH, United States). MATLAB codes were generated to calculate SD1 and SD2 (Poincare plot) as well as the sympathetic Index (SI) using the RR interval signal. The breathing frequency was generated by the Mindware impedance analysis software.

### HRV Related to Posture Change

To test general autonomic reactivity using a standard method, heart rate and HRV changes of all participants were measured related to posture change. The participants refrained from smoking, caffeine intake and heavy eating for 2 h prior to the testing. During the test, they were accommodated in a quiet room with normal lighting and room temperature. After resting in supine position for a minimum 10 min, the ECG and impedance were recorded for 6 min in the supine position, 6 min in sitting position and immediately upon standing for 6 min. The HRV parameters tested are shown in Table 1. We calculated the Baevsky’s Stress Index (SI) (Baevsky and Chernikova, 2017) according to the formula

$$SI=\frac{AM_{o}\times 100\%}{2M_{o}\times M_{x}DM_{n}}$$

**TABLE 1:** Autonomic reactivity associated with posture change.

	Supine Mean ± SEM	Supine Mean ± SEM	*p*-value	(*t, df*) (*t*-test)/ *rs* (Wilcoxon)
RSA [ln(ms)]	6.76 ± 0.28	5.80 ± 0.32	***0.0006	*t* = 4.958, *df* = 10
RMSSD (ms)	57.90 ± 3.65	28.49 ± 3.65	***0.001	*rs* = 0.7671
SD1 (ms)	58.77 ± 6.85	29.53 ± 2.64	**0.0012	*t* = 4.459, *df* = 10
SD2 (ms)	102.94 ± 12.52	82.99 ± 4.77	0.1434	*t* = 1.588, *df* = 10
HF Power (ms^2^)	1552.88 ± 537.25	498.55 ± 120.61	**0.0020	*rs* = 0.7455
LF Power (ms^2^)	1064.53 ± 417.59	1122.36 ± 240.33	0.5771	*rs* = 0.1182
PEP (ms)	121.64 ± 4.94	122.75 ± 6.40	0.8772	*t* = 0.1585, *df* = 10
SI (s^–2^)	32.85 ± 6.96	50.73 ± 5.72	*0.0322	*rs* = 0.4455
LF/HF Ratio	0.69 ± 0.13	3.19 ± 0.64	**0.0049	*rs* = −0.02727
SD2/SD1	1.77 ± 0.07	2.97 ± 0.21	***0.0004	*t* = 5.196, *df* = 10
SI/RSA	5.20 ± 1.21	9.33 ± 1.30	*0.0244	*rs* = 0.5727
SI/RMSSD	0.80 ± 0.22	2.38 ± 0.47	**0.0020	*rs* = 0.5982
HR (bpm)	63.78 ± 2.68	80.50 ± 3.24	*** <0.0001	*t* = 9.854, *df* = 10

*Number of subjects N = 11; t-value and df are reported when the t-test was applied while the rs (Spearman) value is reported where the non-parametric Wilcoxon signed rank test was applied. Abbreviations in this all and other tables: RSA, Respiratory Sinus Arrythmia; SI, Baevsky’s Stress Index or Sympathetic Index; RMSSD, Root Mean Square of Successive Differences; PEP, Pre-Ejection Period; HF, High Frequency; LF, Low Frequency; SD1, Standard deviation of minor axis of Poincare’ Plot; SD2, Standard deviation of major axis of Poincare’x Plot; HR, heart rate.*

where the mode (*M*_*o*_) is the most frequent RR interval expressed in seconds. The amplitude of mode (*AM*_*o*_) was calculated, using a 50 ms bin width, as the number of the RR intervals in the bin containing the *M*_*o*_, expressed as a percentage of the total number of intervals measured. The variability is reflected in *M_*x*_DM_*n*_* as the difference between longest (M*_*x*_*) and shortest (M*_*n*_*) RR interval values, expressed in seconds. The SI is expressed as s^–2^.

### HRV Related to Colonic Motor Patterns

Raw data were obtained from a study that was reported on previously (Milkova et al., 2020; Yuan et al., 2020). High-Resolution Colonic Manometry was performed using an 84-sensor water perfused catheter that detected luminal pressures at 1 cm intervals from the proximal colon to the anal sphincter. The catheter was custom-made by Mui Scientific (Mississauga, ON, Canada) and the acquisition hardware was made by Medical Measurement Systems (Laborie, Toronto, ON, Canada). The sampling frequency of the system is 10 Hz. After the catheter was placed inside the colon with the assistance of colonoscopy, a 6–8 h high-resolution colonic manometry (HRCM) procedure was executed. All participants underwent synchronized HRCM, ECG, and impedance recording during 90 min of baseline, followed by 20 min of proximal balloon distention, 20 min of rectal balloon distention using a standard anorectal manometry balloon assembly, 90 min following intake of a meal, consisting of organic yogurt fortified by organic milk fat to make it 1,000 kcal (Mapleton Organic, ON, Canada), and 45 min after administration of rectal bisacodyl. Participants were supine during all recordings except during the actual intake of the meal.

The manometric analysis was carried out in ImageJ and MATLAB. All High-Amplitude Propagating Pressure Waves (HAPWs) with or without an associated SPW, occurring as single isolated events (Chen et al., 2017) were included in the present study; the motor pattern needed to have a 2 min quiet period before and after the motor pattern. All analysis for the present study was based only on raw data from our studies. Autonomic reactivity to HAPWs was identified by comparing the 2 min period prior to the occurrence of an HAPW, during the occurrence of an HAPW, and the first 2 min immediately after the HAPW. The HRV signal was divided into segments of 1 min and the HRV parameters were calculated for each individual segment. Even if the HAPW lasted 50 sec, the whole segment of 1 min was taken into account. In case of before and after, where the time period taken into account was 2 min, the data was analyzed for each minute separately (using a 1 min window) and the mean of the results of the two segments was taken to represent the HRV parameter. RSA, RMSSD, HF power and SD1 were calculated as measures of parasympathetic activity and LF power, PEP and SI were calculated as measures of sympathetic activity. LF/HF, SD2/SD1 and SI/RSA and SI/RMSSD ratios were also calculated for each phase.

### Analysis of HRV in Association With Motor Complexes

**FIGURE 1:**
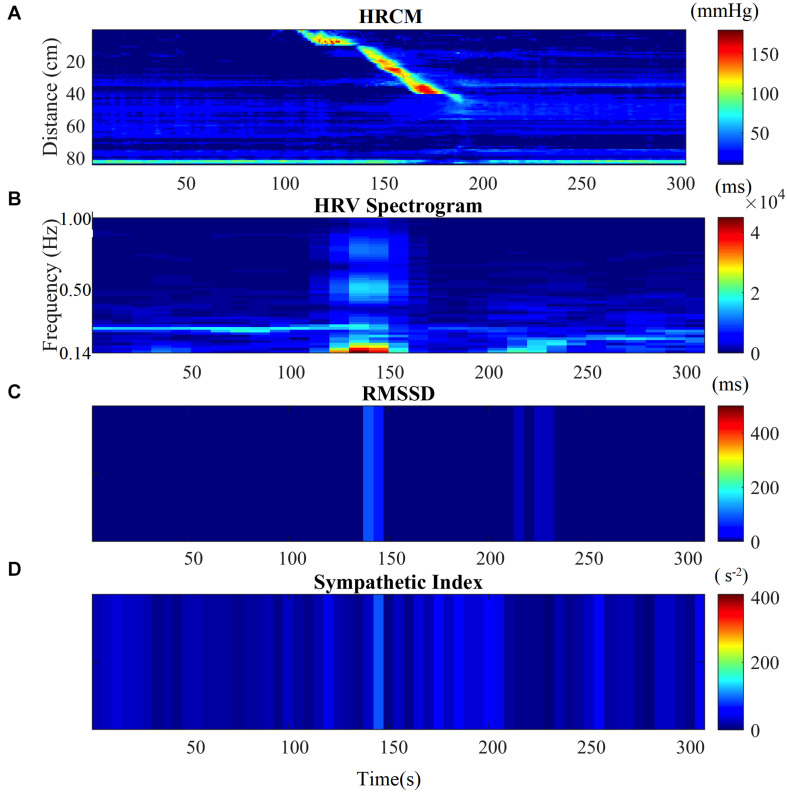
HRV parameters associated with a single HAPW. **(A)** 2-min before, during, and 2-min after HAPW recorded by HRCM. **(B)** HF power/RSA band of HRV signal time matched with HRCM recording. **(C)** RMSSD time matched with HRCM. **(D)** SI time matched with HRCM. Distance at 0 cm is positioned at the proximal colon, distance at 80 cm is just proximal to the anal sphincter.

Autonomic activity related to motor complexes, more than one HAPW as a cluster, was assessed graphically by generating time matched images of the motor complexes in HRCM with the frequency domain HF band (the RSA band) images of the HRV data. The process of generating the HF power (RSA band power) image started by importing the ECG and impedance signal into ImageJ using the Cardio Images plugins (Parsons, 2019). In the Cardio Images plugin, the peak detection and correction of the ECG signal was carried out by a Pan-Tomkins algorithm as well as by a Neural Networks model generated and trained in TensorFlow, followed by manually checking and editing the wrongly detected/edited R peaks. The tachogram of RR intervals was plotted as a raster image using a sampling frequency of 10 Hz, image width of 5 s with cubic interpolation in Intervals plugin. The Frequency Win Plugin was used to calculate FFT spectra of the tachogram raster image using window length of 60 s and intervals of 10 s. The power spectra are collated into an image with time on the y-axis and frequency on the x-axis with pixel intensity as amplitude (ms). Similarly, the HRCM data was converted into an image using the Event Series plugin in ImageJ. Both the images were then imported, and time matched in MATLAB as shown in Figure 1. A Win frequency plugin generated the HRV spectrogram from 0 to 5 Hz, to study the RSA/HF band only; the lower frequency band (0–0.14 Hz.) as well as the frequency band above 1Hz was removed in MATLAB, and the spectrogram with the frequency band of 0.14–1 Hz. was plotted as an aligned image with the HRCM image as shown in Figure 1B. Similarly, the raster image of RR intervals was imported into Matlab and was used to calculate RMSSD and SI, which were also plotted as aligned images with the HRCM Figures 1C,D.

### Statistical Analysis

#### Supine to Standing

To evaluate HRV related to posture change, all the HRV parameters were analyzed independently. Each HRV parameter was calculated for supine and standing position for all the participants (*n* = 11) and tested for normal distribution using the Shapiro-Wilk Normality test. If the data for both supine and standing was normally distributed, the comparison was carried out using the paired *t*-test. The Wilcoxon Matched Pair Signed Rank test was used in case one or both of the supine and standing HRV parameter data was not distributed normally. The change in each HRV parameter was considered significant between supine and standing, if the calculated *p*-value was less than 0.05.

#### High-Resolution Colonic Manometry (HRCM)

All HAPWs (*n* = 65) that had a 2 min period before and after without major motor patterns, in order to obtain a “baseline” and “recovery” period, from all the participants were investigated. For each HRV parameter, the results from all HAPWs were averaged for each subject and were presented as one reading with three data points (Before-During-After). These averaged results from all the participants were used for further analysis. Initially, the data was tested for normal distribution using the Shapiro-Wilk Normality test. If the data was normally distributed, the parametric test ANOVA followed by Bonferroni Multiple Comparison test was used for comparison. While the non-parametric Friedman test followed by Dunn’s Multiple Comparison test was used for data sets that were not normally distributed. The *p*-value was calculated for before-to-during [*p*-value (B-D)] and during-to-after [*p*-value (D-A)]. A difference was considered significant when *p* < 0.05. The *t*-values and degrees of freedoms are reported with parametric tests while the *z*-value is reported with non-parametric tests.

In addition, the HAPW’s were grouped based on the HRCM condition with 12 HAPW’s observed during baseline, 16 during meal, 14 during prucalopride, 5 during proximal balloon distension, 7 during distal balloon distension and 11 during bisacodyl. The same statistical procedures as mentioned above were applied to each group separately to identify the effect of the stimulus conditions on the association of autonomic nervous system with HAPW’s.

## Results

### Autonomic Reactivity Associated With Posture Change

The parasympathetic parameters RSA, RMSSD, SD1, and HF power all decreased from supine to standing consistent with a decrease in parasympathetic reactivity. The sympathetic parameter SI showed a significant increase from supine to standing. PEP did not show any significant change. The shift from parasympathetic to sympathetic going from supine to standing was reflected in the change in LF/HF ratio, SD2/SD1 ratio, SI/RSA as well as the SI/RMSSD ratio. The posture change resulted in an increase in heart rate. SD2 and LF power did not change, likely a reflection of the fact that these parameters are associated with both sympathetic and parasympathetic changes (Table 1).

### Autonomic Reactivity Associated With HAPWs

A significant increase in RSA indicated activation of the parasympathetic nervous system during the motor activity as compared to the period before the motor pattern and the change recovered within 2 min (Table 2). An increase in RSA during the HAPWs was seen in all subjects, average 9.3%, with recovery afterward. Similarly, an increase in RMSSD was seen in all subjects except one. There was an average increase of 24.6% in the RMSSD during the HAPW (Table 2). Due to one outlier, the change in RMSSD did not reach statistical significance. Similarly, SD1 also increased numerically in all volunteers but one, and did not reach statistical significance (Table 2).

**TABLE 2:** Autonomic nervous system modulation in association with all individual HAPWs and HAPW-SPWs combined.

	Before Mean ± SEM	During Mean ± SEM	After Mean ± SEM	*p*-value (B-D) (*t, df*) or (*z*-value)	*p*-value (D-A) (*t, df*) or (*z*-value)
RSA [ln(ms)]	6.38 ± 0.27	6.90 ± 0.26	6.46 ± 0.24	*0.0182 (3.420, 8)	*0.0220 (3.290, 8)
RMSSD (ms)	53.24 ± 6.73	66.35 ± 10.35	52.03 ± 8.34	0.1609 (1.862, 8)	0.1608 (1.973, 8)
SD1 (ms)	45.19 ± 4.66	56.44 ± 6.93	40.93 ± 4.42	0.1201 (2.189, 8)	0.0593 (2.641, 8)
SD2 (ms)	100.02 ± 7.64	126.93 ± 12.76	79.44 ± 0.18	*0.0069 (4.094, 8)	*0.0166 (3.481, 8)
HF Power (ms^2^)	1675.88 ± 715.99	1594.33 ± 425.33	1074.31 ± 319.33	0.1979 (1.650)	0.1187 (1.886)
LF Power (ms^2^)	1477.46 ± 241.43	1223.29 ± 200.60	588.49 ± 143.83	0.8796 (0.8128, 8)	*0.0128 (3.661, 8)
PEP (ms)	115.04 ± 3.02	114.91 ± 4.14	118.06 ± 7.25	>0.9999 (0.053, 7)	0.4309 (1.362, 7)
SI (s^–2^)	94.9 ± 29.1	51.92 ± 18.43	88.81 ± 25.62	*0.0190 (2.593)	**0.0044 (3.064)
LF/HF Ratio	2.75 ± 0.75	1.21 ± 0.24	0.88 ± 0.20	0.4772 (1.179)	0.1187 (1.886)
SD2/SD1	2.62 ± 0.19	2.53 ± 0.23	2.15 ± 0.18	>0.9999 (0.00)	0.1542 (1.768)
SI/RSA	18.76 ± 6.92	8.69 ± 3.69	16.13 ± 5.27	**0.0094 (2.828)	***0.0008 (3.536)
SI/RMSSD	5.53 ± 2.60	1.97 ± 1.24	3.52 ± 1.72	**0.0094 (2.828)	***0.0008 (3.536)
HR (bpm)	69.39 ± 3.98	66.73 ± 3.79	64.33 ± 3.64	0.1434 (2.075, 8)	0.4711 (1.283, 8)

The number of subjects, N = 9; the number of HAPW’s, n = 65; t-value and df are reported for the parametric test (ANOVA) and z-value is reported in case of the non-parametric Friedman test. *P ≤ 0.05; **P ≤ 0.01, ***P ≤ 0.001.

Although RSA showed a significant increase, the HF power, derived from the same data set as the RSA, did not show a significant change (Table 2). RSA is the natural log (*ln*) of HF power and taking a natural log will remove the effect of large outliers. Indeed, when we removed 4 out of 65 values from the HF power data set that showed more than 3 SD units off the mean value, the HF power changed from 993.07 ± 300.47 before the motor pattern to 1769.64 ± 546.76 (*p* = 0.0485) and recovered to 1135.88 ± 403.85 (*p* = 0.0485); the increase in RMSSD during the HAPW and its decrease afterward, also became significant.

The change in sympathetic index (SI) indicated a decrease in sympathetic activity during the motor patterns that recovered within 2 min (Table 2). The PEP did not show significant changes (Table 2).

The SI/RSA decreased 42% during an HAPW and recovered within 2 min, consistent with activity shifting toward parasympathetic activity during the motor activity. Similarly, SI/RMSSD showed a 64.4% decrease. Both the LF/HF and SD2/SD1 ratios did not change significantly with motor activity. The heart rate did not show any significant change with motor activity (Table 2).

Since RSA is sensitive to respiratory rate changes and to respiratory tidal volume changes, the breathing frequency and volume were calculated before during and after all HAPWs. The breathing frequencies before and during all HAPWs, were 15.9 ± 0.4 and 15.4 ± 0.5 breaths/min (*P* = 0.225), and 15.20 ± 0.47 (*p* > 0.9999) after the HAPW. The values for volume were 0.0123 ± 0.0113 and 0.0131 ± 0.0008 V^2^ (*P* > 0.999), respectively, and it was 0.008 ± 0.007 V^2^ (*p* = 0.7420) after the HAPW. Hence, no significant change in breathing frequency was observed in response to an HAPW.

Autonomic activity associated with HAPWs may arise from the activity that initiates the HAPW and from potential mechanoreceptors activated by the actual HAPW. Although rectal bisacodyl almost always evoked HAPWs in the present cohort of healthy subjects, in one subject, two low amplitude simultaneous pressure waves were associated with an increase in RSA from 4.83 to 5.88 *ln(ms)* with a concomitant decrease in SI from 159 to 84 s^–2^; consistent with the notion that the initiating autonomic activity is seen by HRV and that the change may not solely dependent on the strong HAPW evoking distention.

### Autonomic Nervous System Associations With HAPW’s in the Different Conditions

Activity of autonomic nervous system activity during an HAPW may be different in different conditions, hence we assessed HRV parameter changes separately under each condition: baseline, meal, prucalopride, proximal balloon distension, distal balloon distension and bisacodyl, The dramatic shift in autonomic balance toward a dominant parasympathetic activity that was described above was observed during the HAPWs that were evoked by a meal (Table 3A) and by rectal bisacodyl (Table 3B) as reflected by RSA, RMSSD, SI and SI/RSA and SI/RMSSD.

**TABLE 3A:** HRV parameters associated with HAPWs in response to the meal (*n* = 16).

	Before ± SEM	During ± SEM	After ± SEM	*p*-value (B-D) (*t, df*) or (*z*-value)	*p*-value (D-A) (*t, df*) or (*z*-value)
RSA [ln (ms)]	6.22 ± 0.24	6.72 ± 0.20	6.21 ± 0.17	*0.0473 (2.54, 14)	**0.0013 (4.47, 13)
RMSSD (ms)	48.15 ± 10.45	47.93 ± 6.90	38.35 ± 5.13	*0.0352 (2.37)	**0.0038 (3.10)
SD1 (ms)	40.24 ± 6.68	46.34 ± 4.85	35.53 ± 4.69	0.0569 (2.19)	**0.002 (3.29)
SD2 (ms)	94.45 ± 15.96	112.38 ± 10.387	79.72 ± 9.3107	*0.0352 (2.37)	*0.0212 (2.56)
HF Power (ms^2^)	1930.41 ± 1100.6	1199.00 ± 295.64	641.37 ± 112.73	0.1358 (1.83)	**0.0038 (3.10)
LF Power (ms^2^)	1239.22 ± 405	1296.89 ± 355.38	667.15 ± 135.33	0.4025 (1.28)	**0.0212 (2.56)
PEP (ms)	120.91 ± 2.29	123.27 ± 2.36	124.00 ± 1.72	0.2575 (1.58, 13)	0.9107 (0.11, 13)
SI (s^–2^)	77.34 ± 10.17	55.30 ± 7.01	89.57 ± 12.22	*0.0467 (2.27)	**0.0092 (2.84)
LF/HF Ratio	2.06 ± 0.84	1.26 ± 0.27	1.43 ± 0.37	>0.9999 (0.36)	0.9304 (0.73)
SD2/SD1	2.67 ± 0.60	2.40 ± 0.22	2.63 ± 0.34	0.4025 (1.79)	>0.9999 (0.36)
SI/RSA	11.60 ± 1.82	8.12 ± 1.27	13.87 ± 2.03	*0.0123 (2.78)	**0.002 (3.29)
SI/RMSSD	2.33 ± 0.44	1.48 ± 0.32	2.69 ± 0.46	*0.0123 (2.74)	**0.002 (3.29)
HR (bpm)	71.50 ± 1.96	70.33 ± 1.80	70.35 ± 1.80	>0.9999 (0.18)	>0.9999 (0.09)

t-value and df are reported for parametric tests (ANOVA) and the z-value is reported with non-parametric Friedman tests. *P ≤ 0.05; **P ≤ 0.01.

**TABLE 3B:** HRV parameters associated with HAPWs in response to rectal bisacodyl (*n* = 11).

	Before ± SEM	During ± SEM	After ± SEM	*p*-value (B-D) (*t, df*) or (*z*-value)	*p*-value (D-A) (*t, df*) or (*z*-value)
RSA [ln (ms)]	5.67 ± 0.57	6.13 ± 0.48	5.68 ± 0.47	*0.0407 (2.35, 10)	0.2529 (1.23, 8)
RMSSD (ms)	32.95 ± 7.45	46.00 ± 9.52	33.79 ± 7.90	***0.0007 (3.58)	*0.0278 (2.46)
SD1 (ms)	26.87 ± 4.38	36.90 ± 6.70	22.48 ± 4.22	*0.0268 (2.99, 10)	**0.0582 (2.21, 8)**
SD2 (ms)	75.42 ± 9.70	78.24 ± 7.89	62.71 ± 8.77	0.2896 (1.12, 10)	0.1159 (2.19, 8)
HF Power (ms^2^)	694.90 ± 226.5	1037.75 ± 296.52	544.23 ± 142.21	0.3594 (1.34)	0.0883 (2.01)
LF Power (ms^2^)	1181.99 ± 409.5	1079.44 ± 231.31	465.79 ± 108.27	0.7422 (0.98)	*0.0278 (2.46)
PEP (ms)	108.00 ± 3.25	109.23 ± 3.19	111.11 ± 3.00	0.5032 (1.09, 13)	0.5032 (1.09, 9)
SI (s^–2^)	222.29 ± 75.10	146.39 ± 58.42	223.09 ± 72.76	*0.0278 (2.46)	**0.0073 (2.91)
LF/HF Ratio	2.57 ± 0.49	2.21 ± 0.80	1.36 ± 0.42	0.5271 (1.12)	0.5271 (1.12)
SD2/SD1	2.95 ± 0.31	2.44 ± 0.22	2.68 ± 0.43	0.2544 (1.59, 13)	0.6402 (0.48, 9)
SI/RSA	54.98 ± 21.95	29.75 ± 13.02	45.09 ± 15.53	*0.0146 (2.68)	**0.0016 (3.35)
SI/RMSSD	23.32 ± 7.98	11.53 ± 4.61	18.85 ± 6.58	**0.0094 (2.83)	**0.0094 (2.83)
HR (bpm)	86.22 ± 5.18	84.00 ± 5.50	82.98 ± 4.93	0.0883 (2.01)	>0.9999 (0.34)

t-value and df are reported for parametric tests (ANOVA) and the z-value is reported with non-parametric Friedman tests. *P ≤ 0.05; **P ≤ 0.01, ***P ≤ 0.001.

During baseline, the mean values of all the HRV parameters during HAPW changed in the expected direction (5.03% increase in RSA, 6.53% increase is RMSSD, 24.79% increase in SD1, 38.68% increase in HF power, 30.44% decrease in SI), but the changes did not reach statistical significance (Table 3C).

**TABLE 3C:** HRV parameters associated with HAPWs during baseline (*n* = 12).

	Before ± SEM	During ± SEM	After ± SEM	*p*-value (B-D) (t, df) or (*z*-value)	*p*-value (D-A) (*t, df*) or (*z*-value)
RSA [ln (ms)]	7.01 ± 0.30	7.37 ± 0.33	6.81 ± 0.33	0.6419(1.25,21)	0.0695(1.91,21)
RMSSD (ms)	72.23 ± 9.83	76.94 ± 11.77	60.62 ± 9.37	> 0.9999(0.43)	0.066 (2.12)
SD1 (ms)	64.65 ± 7.83	80.68 ± 8.91	66.18 ± 7.31	0.0562(2.31,11)	**0.0086 (3.67, 10)
SD2 (ms)	111.94 ± 8.06	152.09 ± 10.06	110.47 ± 9.33	*0.0136 (2.93, 11)	**0.001 (5.06, 10)
HF Power (ms^2^)	1964.75 ± 519.63	2724.68 ± 1146.1	1766.25 ± 629.52	> 0.9999(0.21)	0.5728 (1.06)
LF Power (ms^2^)	1649.09 ± 397.7	1629.77 ± 369.21	970.19 ± 402.49	> 0.9999(0.61)	> 0.9999(0.61)
PEP (ms)	120.50 ± 2.41	125.33 ± 2.11	125.45 ± 2.26	0.1181(2.09,11)	0.9486(0.07,10)
SI (s^–2^)	34.00 ± 5.89	23.65 ± 3.41	57.88 ± 12.46	0.3316 (1.39)	**0.004 (3.09)
LF/HF Ratio	2.04 ± 0.77	1.08 ± 0.30	1.04 ± 0.23	0.7845 (0.85)	> 0.9999(0.21)
SD2/SD1	2.04 ± 0.25	2.11 ± 0.23	1.83 ± 0.19	0.7648(0.31,11)	0.2662(1.59,10)
SI/RSA	4.99 ± 0.94	3.35 ± 0.52	9.27 ± 2.25	0.4017 (1.28)	**0.0028 (3.20)
SI/RMSSD	0.63 ± 0.16	0.39 ± 0.08	1.39 ± 0.41	0.2712 (1.49)	***0.0006 (3.62)
HR (bpm)	56.92 ± 2.18	54.42 ± 1.93	54.36 ± 1.50	0.0897(2.25,11)	0.9206(0.10,10)

t-value and df are reported for parametric tests (ANOVA) and the z-value is reported with non-parametric Friedman tests. *P ≤ 0.05; **P ≤ 0.01, ***P ≤ 0.001.

The 90 min period after oral prucalopride, where we hypothesize that prucalopride stimulates the gastric mucosa to evoke HAPWs as a gastrocolic reflex, both RSA and RMSSD increased significantly, and SI decreased significantly during the HAPW’s. and recovery afterward in both RMSSD and SI was also significant. A significant shift in autonomic balance toward parasympathetic activity was indicated by a decrease in SI/RMSSD (Table 3D).

**TABLE 3D:** HRV parameters associated with HAPWs in response to prucalopride (*n* = 14).

	Before ± SEM	During ± SEM	After ± SEM	*p*-value (B-D) (t, df) or (*z*-value)	*p*-value (D-A) (t, df) or (*z*-value)
RSA [ln (ms)]	6.17 ± 0.23	6.80 ± 0.28	6.42 ± 0.35	*0.0216 (2.55)	0.2333 (1.57)
RMSSD (ms)	43.30 ± 6.16	55.97 ± 7.42	53.95 ± 0.35	*0.0467 (2.27)	*0.0467 (2.27)
SD1 (ms)	35.68 ± 5.253	45.74 ± 6.24	41.2817 ± 7.23	0.1025(2.12,13)	0.2165(1.30,13)
SD2 (ms)	76.897925 ± 5.93	93.16 ± 9.52	82.4542 ± 10.48	0.0731(2.32,13)	0.0731(1.98,13)
HF Power (ms^2^)	694.95 ± 205.84	1327.93 ± 351.56	1304.46 ± 472.90	0.0997 (1.96)	0.6536 (0.98)
LF Power (ms^2^)	595.53 ± 119.8	911.97 ± 274.02	607.13 ± 242.66	> 0.9999(0.39)	0.5615 (1.08)
PEP (ms)	110.67 ± 3.74	113.33 ± 3.62	115.17 ± 3.60	0.23(1.65,13)	0.2692(1.15,13)
SI (s^–2^)	91.06 ± 12.60	65.34 ± 11.87	87.27 ± 19.55	*0.0465 (2.56, 13)	*0.0465 (2.34, 13)
LF/HF Ratio	1.20 ± 0.23	0.75 ± 0.10	0.64 ± 0.16	0.2333 (1.57)	> 0.9999(0.39)
SD2/SD1	2.478 ± 0.27	2.275 ± 0.26	2.31167 ± 0.32	0.8994 (0.76)	> 0.9999(0.38)
SI/RSA	15.17 ± 2.15	10.43 ± 2.17	15.00 ± 3.62	0.2611 (1.51)	0.1176 (1.89)
SI/RMSSD	2.94 ± 0.45	1.65 ± 0.37	3.63 ± 1.09	*0.0467 (2.27)	*0.0092 (2.84)
HR (bpm)	71.75 ± 4.19	70.33 ± 4.01	70.07 ± 3.64	> 0.9999(0.38)	> 0.9999(0.19)

t-value and df are reported for parametric tests (ANOVA) and the z-value is reported with non-parametric Friedman tests. *P ≤ 0.05.

The periods of balloon distention had low n numbers, nevertheless, distal balloon distension was accompanied by a significant increase in RSA and recovery after the HAPW (Table 3E), but changes in response to proximal balloon distention did not reach significance (Table 3F).

**TABLE 3E:** HRV parameters associated with HAPWs in response to distal balloon distention (*n* = 7).

	Before ± SEM	During ± SEM	After ± SEM	*p*-value (B-D) (*t, df*) or (*z*-value)	*p*-value (D-A) (*t, df*) or (*z*-value)
RSA [ln (ms)]	6.56 ± 0.41	7.31 ± 0.20	6.25 ± 0.21	*0.0138 (4.42, 5)	*0.0008 (10.88, 4)
RMSSD (ms)	51.73 ± 8.29	61.19 ± 8.43	43.51 ± 3.59	0.7593(1.85,5)	0.4863(1.35,4)
SD1 (ms)	52.82 ± 10.39	49.69 ± 5.95	42.03 ± 4.57	0.7649(0.31,6)	0.7351(0.77,4)
SD2 (ms)	105.08 ± 11.97	120.76 ± 13.77	90.732 ± 11.20	0.2277 (1.58)	0.4118 (1.26)
HF Power (ms^2^)	1449.08 ± 561.1	1844.86 ± 344.43	631.55 ± 162.59	> 0.9999(0.53,5)	*0.0493 (4.20, 3)
LF Power (ms^2^)	842.96 ± 438.2	1574.24 ± 370.03	520.67 ± 212	0.3146 (1.41)	0.1542 (1.77)
PEP (ms)	123.43 ± 2.09	126.00 ± 2.68	127.60 ± 1.91	0.6296(0.92,6)	0.638(0.51,4)
SI (s^–2^)	48.39 ± 10.42	43.67 ± 7.95	61.04 ± 12.76	0.7077(0.39,6)	0.6823(0.86,4)
LF/HF Ratio	7.92 ± 2.02	5.88 ± 0.94	10.06 ± 2.35	0.5777 (1.06)	0.5777 (1.06)
SD2/SD1	2.594 ± 0.47	3.31 ± 1.15	2.21 ± 0.28	> 0.9999(0.32)	> 0.9999(0.63)
SI/RSA	7.92 ± 2.02	5.88 ± 0.94	10.06 ± 2.35	0.4536(0.88,6)	0.4536(1.31,4)
SI/RMSSD	1.30 ± 0.43	0.79 ± 0.13	1.52 ± 0.40	0.5194(1.24,6)	0.5168(1.32,4)
HR (bpm)	63.29 ± 1.70	64.14 ± 2.06	62.31 ± 1.07	0.6954(0.81,6)	0.6954 (0.76.4)

t-value and df are reported for parametric tests (ANOVA) and the z-value is reported with non-parametric Friedman tests. *P ≤ 0.05.

**TABLE 3F:** HRV parameters associated with HAPWs in response to proximal balloon distention (*n* = 5).

	Before ± SEM	During ± SEM	After ± SEM	*p*-value (B-D) (*t, df*) or (*z*-value)	*p*-value (D-A) (*t, df*) or (*z*-value)
RSA [ln (ms)]	6.63 ± 0.36	6.75 ± 0.18	7.10 ± 0.31	> 0.9999(0.33,4)	0.6283(1.18,4)
RMSSD (ms)	46.44 ± 0.36	43.99 ± 5.85	48.88 ± 3.65	> 0.9999(0.45,5)	> 0.9999(0.64,4)
SD1 (ms)	39.03 ± 2.91	40.57 ± 7.04	46.17 ± 4.60	0.7593(0.27,5)	0.4863(0.66,4)
SD2 (ms)	87.99 ± 14.16	85.74 ± 9.28	100.57 ± 11.71	> 0.9999(0.32)	> 0.9999(0.32)
HF Power (ms^2^)	805.16 ± 293	818.90 ± 134.34	1119.5 ± 444.78	> 0.9999(0.05,5)	> 0.9999(0.60,4)
LF Power (ms^2^)	1347.52 ± 529.7	601.13 ± 212.58	879.83 ± 444.92	0.6856 (0.95)	> 0.9999(0.00)
PEP (ms)	118.33 ± 3.38	111.33 ± 6.35	118.40 ± 3.68	0.2225(1.88,5)	0.2225(1.87,4)
SI (s^–2^)	61.36 ± 7.67	67.27 ± 15.99	57.17 ± 10.36	0.9121(0.40,5)	0.9121(0.35,4)
LF/HF Ratio	10.20 ± 1.87	10.19 ± 2.38	8.24 ± 1.60	0.3095 (1.42)	> 0.9999(0.47)
SD2/SD1	2.28 ± 0.32	2.34 ± 0.31	2.39 ± 0.49	0.993(0.11,5)	0.993(0.076,4)
SI/RSA	10.20 ± 1.87	10.19 ± 2.38	8.24 ± 1.60	0.9942(0.01,5)	0.8978(0.44,4)
SI/RMSSD	1.16 ± 0.21	1.61 ± 0.57	1.23 ± 0.25	0.7325(1.02,4)	> 0.9999(0.45,4)
HR (bpm)	65.00 ± 2.75	65.50 ± 3.45	64.58 ± 3.77	0.8425(0.31,5)	0.8425(0.56,4)

*t-value and df are reported for parametric tests (ANOVA) and the z-value is reported with non-parametric Friedman tests.*

### Autonomic Reactivity Associated With Motor Complexes

**FIGURE 2:**
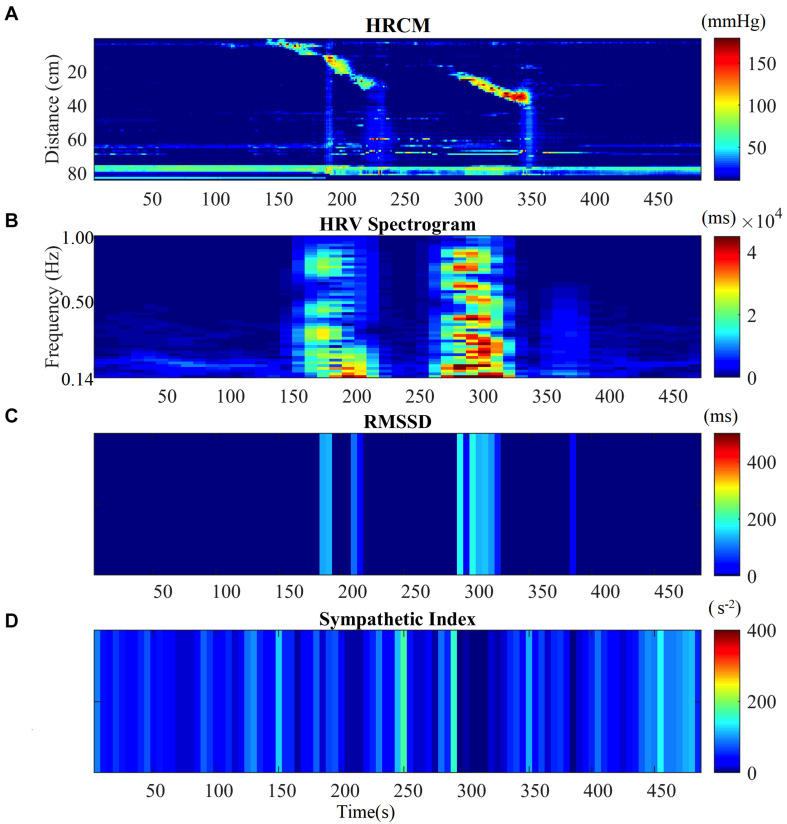
HRV parameters associated with two consecutive HAPWs. **(A)** Before-During and After a Motor Complex. **(B)** Time matched HF Power/RSA band of the HRV signal before-during and after MC. **(C)** RMSSD time matched with HRCM. **(D)** SI time matched with HRCM.

**FIGURE 3:**
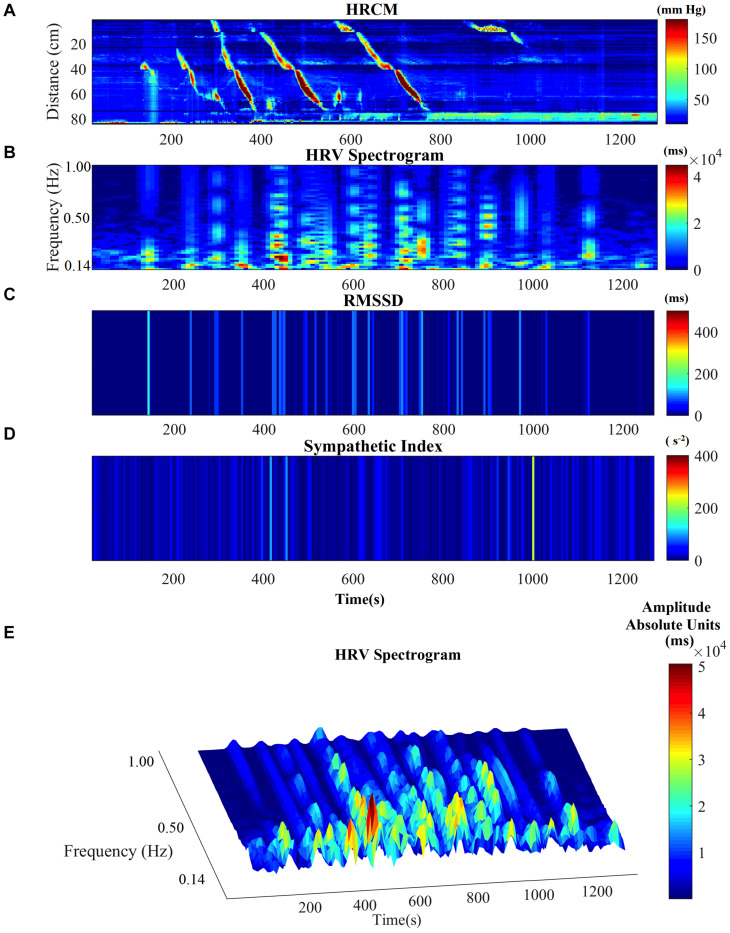
HRV parameters associated with a complex of multiple HAPWs (a motor complex). **(A)** HRCM recording with a motor complex containing long overlapping HAPWs. **(B)** HF power/RSA band of HRV signal. **(C)** RMSSD. **(D)** SI. **(E)** HF/RSA power band in 3D.

**FIGURE 4:**
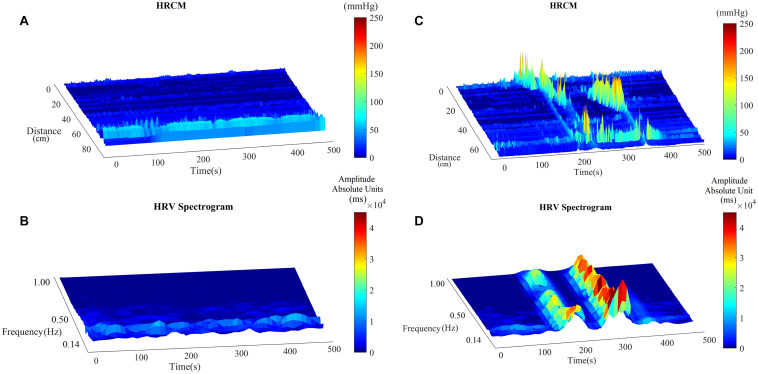
The RSA band in baseline and with HAPWs. **(A)** Baseline HRCM recording without any colonic motor pattern. **(B)** HF power/RSA band during the baseline. **(C)** HRCM recording with Motor Complex. **(D)** HF power/RSA band during the Motor Complex.

Motor complexes are defined here as more than one HAPW and/or HAPW-SPW that occurred close together such that they could not be analyzed separately. In order to assess HRV during the motor complexes, a continuous assessment procedure was developed as outlined in the methods section. The major finding was that motor complexes were associated with an increase in HF power that was not continuous but rhythmic. The average duration of RSA reactivity, measured at 0.14–0.5 Hz, was 50 ± 10 s and the frequency of occurrence was 0.8 ± 0.2 cycles/min which was similar to the HAPW frequency within motor complexes (Figure 2). However, with long HAPWs, more than one RSA band occurred, giving the RSA activity a distinct rhythmic appearance (Figure 3). 37 out of a total 40 motor complexes studied, had RSA bands associated with them. Although there was complete synchronization of individual HAPWs and bursts of RSA activity, with motor complexes (*n* = 34), rhythmic RSA activity sometimes (*n* = 6) continued after the HAPW to slowly die out. Sometimes (*n* = 3), the RSA activity started prior to the measured HAPW, but the HAPW likely originated earlier at a more proximal site, beyond the reach of the catheter. RMSSD also increased during the HAPWs and motor complexes. There was complete synchronization between RSA and RMSSD. SI changes were observed as more or less reciprocal to the RMSSD and RSA bands (Figures 1, 2). During all 90 min baseline periods, when HAPWs are rare, there was never rhythmic HF activity although very low amplitude HF activity was continuously observed (Figure 4).

## Discussion

### Assessment of Sympathetic Activity During Posture Change

In the assessment of sympathetic increase in the supine to standing protocol, the Baevsky Stress Index or Sympathetic Index increased 52%, whereas SD2, PEP and the LF power did not show significant changes. In order to maintain a near constant blood pressure, in response to the postural changes from supine to standing when blood is pooled in the legs and blood pressure decreases, the baroreceptor reflex increases sympathetic activity to increase heart rate. Baroreceptor action potentials are relayed to the nucleus tractus solitarius, which uses action potential frequency as a measure of blood pressure. The end-result of baroreceptor de-activation is excitation of the sympathetic nervous system and inactivation of the parasympathetic nervous system. Houtveen et al. (2005) recorded PEP at different breathing frequencies during supine, sitting and standing; PEP increased from supine to standing hence the sympathetic activity appeared to decrease. This was also observed by Cacioppo et al. (1994). PEP is a measure of ventricular contractility, influenced by beta-adrenergic receptor mediated ventricular sympathetic innervation (Van Lien et al., 2015). Nitroprusside causes vasodilation, baroreceptor unloading and a reflex increase in sympathetic tone, which was associated with a significant decrease in PEP (Schächinger et al., 2001). Our study indicates that the sympathetic activity that is increased upon standing is not reflected in the PEP value.

Sympathetic pathways within the body form a vast network; only those sympathetic activities that interact with, or directly or indirectly take part in sympathetic regulation of heart rate will be seen in HRV. For example, muscle sympathetic nerve activity measured at the peroneal nerve induces vasoconstriction and is modulated by the baroreflex but it represents regional sympathetic neural activity, it does not equal sympathetic discharge directed to the heart (Schächinger et al., 2001).

Both LF power and SD2 did not significantly increase with posture change in the present study. Many studies continue to presume that LF power, especially if adjusted for HF power, total power, or respiration, provides an index of cardiac sympathetic “tone” and that the ratio of LF:HF power indicates “sympathovagal balance,” but strong evidence has been presented that LF power neither reflects cardiac sympathetic tone at supine nor in response to standing (Goldstein et al., 2011). There is also no evidence that the LFa (low frequency area) (Nguyen et al., 2020) is specific for sympathetic activity (Goldstein et al., 2011; Rahman et al., 2011). Rahman et al. (2018) showed that SD2 as well as the ratio of SD1 and SD2 are not related to cardiac sympathetic activity.

Baevsky et al. developed an index of regulation strain, or stress index, or, relevant to our study, a “Sympathetic Index” which illustrates the sympathetic or central regulation activity (Baevsky and Chernikova, 2017). The activation of sympathetic regulation results in the stabilization of the heart rhythm which causes a decrease in variation of RR intervals and an increase in the number of intervals with similar duration. The histogram of RR intervals becomes narrower and increases in height. Although the SI is not yet widely applied for sympathetic measurement, it is used by commercially available HRV data analysis software as one of the measures of sympathetic activity (Kubios, 2020); they use the square root of SI to minimize the effect of outliers. The marked change in SI due to posture change in the present study suggests it to be a sensitive marker for orthostatic sympathetic change.

### Sympathetic Activity and the Colonic Propulsive Motor Patterns

The HAPWs, the most significant propulsive motor pattern of the human colon, were accompanied by a significant decrease in SI, hence a decrease in sympathetic activity. During motor complexes, SI was always showing some value above zero, which indicates that there was continuous sympathetic activity which was inhibited during HAPWs. We infer that withdrawal of sympathetic activity is part of the autonomic reflexes that initiate the HAPW, e.g., in response to a meal or rectal bisacodyl. Could stretch receptors be activated during the HAPW? Viscerofugal neurons, connecting to the sympathetic prevertebral ganglia allow the colon to fill, and when the circular muscle of the colon wall contracts to empty the segment, the mechanoreceptors are unloaded and synaptic input decreases (Szurszewski and Miller, 2006). Hence the marked reduction in SI observed in the present study is consistent with a withdrawal of sympathetic activity, allowing the HAPW to proceed.

### Parasympathetic Activity and the Colonic Propulsive Motor Patterns

All HRV parameters studied that are associated with parasympathetic activity, were decreased in response to posture change from supine to standing reflecting the well-known decrease in parasympathetic activity to prevent orthostatic hypotension.

Individual HAPWs, were associated with a significant increase in RSA. Many of these HAPWs occurred during baseline or in the aftermath of taking a meal where the HAPWs are not felt and do not cause a sensation and are not associated with evoked body movements, discomfort or changes in breathing patterns. We suggest that this may reflect the activity in the parasympathetic nervous system associated with the initiation of the HAPW. HAPWs occur most often after a meal as a result of the gastro-colonic reflex, or in response to rectal stimulation where they are the result of a sacral defecation reflex. The sacral defecation reflex involves the sacral defecation center (the parasympathetic nucleus), Barrington’s nucleus and the NTS (Taché and Million, 2015). Barrington’s nucleus and the NTS are also involved in cardiac homeostasis (Gasparini et al., 2020), and in this way, the neuronal traffic in association with HAPWs can influence heart rate, similar to the influence of breathing on the heart rate: “breathing at different rates within the 9–24 bpm range, which changes HF power, does not change mean heart rate” (Shaffer and Ginsberg, 2017). The fact that a significant change occurred in RSA in association with the motor patterns without a change in heart rate is consistent with the hypothesis that the origin of the parasympathetic activity is the neural activity associated with gut activity and not cardiac activity. The fact that the heart rate does not change suggests that the vagal tone, the mean vagal efferent effects to the sinus node, does not change (Grossman and Taylor, 2007).

Vagal afferent neurons are likely activated by colonic motor patterns and their dendritic projections extent throughout the NTS and intermingle within the subnuclei providing a potential means to coordinate respiratory and cardiac autonomic activities (Browning and Travagli, 2014). Sensory nerves in the pelvic plexus will also convey colonic information to the spinal cord (Smith-Edwards et al., 2019). Hence neuronal traffic originating for an HAPW may also influence HRV. Research is ongoing to distinguish efferent and afferent neuronal traffic associated with HAPWs and to explore their role in HRV changes and diagnostic value. It is also possible that HAPWs induce colonic blood pressure changes that might influence HRV (Semba and Fujii, 1970).

When HAPWs occur in quick succession within motor complexes, overlapping in time, RSA was markedly increased. Strong motor patterns induced by bisacodyl sometimes results in discomfort and increased breathing frequency; increased breathing frequency results in decreased RSA but this was not found, likely superseded by processes that increased RSA. Importantly, there was never continuous RSA activity even when HAPWs were continuously present. RSA increases occurred in bursts that were not synchronous with individual HAPWs within the burst, and they continued for several minutes and then diminished gradually (Figure 3). The HF “rhythmicity” suggests that there is a refractory period in the parasympathetic neural activity. In some instances, the RSA bands started prior to the HAPW’s, however, in those cases the HAPWs were seen in the most proximal sensor hence the true beginning of the HAPW was not captured by the catheter. Since contractions are ongoing, the rhythmicity of the parasympathetic activity suggests that it arises from activity orchestrating the HAPWs and not from distention, but this remains to be investigated.

### HRV Parameters Associated With Autonomic Reflexes

When the HRV parameters associated with HAPWs were analyzed within each intervention separately, strong associations between HAPWs and sympathetic decrease and parasympathetic increase were observed in response to a meal, which reflect the gastrocolic reflex, in response to rectal bisacodyl that reflect the sacral autonomic (defecation) reflex and in a rapid response to oral prucalopride which we hypothesize is due to a gastrocolic reflex mediated by stimulation of gastric enterochromaffin cells and subsequent activation of vagal sensory nerves. In a subset of patients with chronic constipation, absence of the autonomic reflexes is associated with high sympathetic activity (Chen and Liu, unpublished observations).

### RSA vs. RMSSD and HF Power

The RMSSD increased one to one with the increase in RSA associated with HAPWs and within the motor complexes, confirming the marked association between parasympathetic activity and human colon propulsive motor patterns. The association between RMSSD and single HAPWs occurred despite the fact that the recording period of 1 min during the HAPW was too short for an ideal assessment as argued by Baek et al. (2015).

The RSA is the natural log of the HF power; the HF power did not show a significant change likely because with a few HAPWs (4/65), the HF power was more than 3 standard deviations from the mean values. If we took these values out and assessed 61 out of 65 motor patters, the HF power showed a significant increase going from baseline to motor pattern and back to recovery.

### The Significance of Changes in Breathing Frequency

The HF band, also known as the respiratory band, is associated with variation in heart rate due to respiration. The HF band has been set to range from 0.15 to 0.4 Hz. which corresponds to the respiration frequency of 9–24 breaths/min, the normal frequency range in adults. The heart rate increases during inhalation due to inhibition of vagal outflow and decreases during exhalation due to the restoration of vagal outflow by release of acetylcholine (Gasparini et al., 2020). If the breathing rate is outside the range of 9–24 breaths/min, then the calculated HF power will be due to noise or harmonics of lower frequency bands and the respiration frequency power will not be included in the HF power. If the respiration frequency is lower than 9 breaths/min, as is the case with slow, deep breathing, the most prominent component of the respiration frequency power will lie in the LF band, and without adjusting the HF band, it will indicate low HF power and high LF power and lead to wrong interpretations of HF power. Similarly, during exercise, a breathing frequency of over 24 breaths/min, the prominent component of parasympathetic power will be out of the range and will wrongly represent low parasympathetic power. If during experimental conditions the breathing frequency goes outside the 9–24 breaths/min range, then the HF power band can be adjusted based on the respiration frequency. This was used in a recent study by Nguyen et al. where the HF or parasympathetic band was replaced by “RFa” (respiratory frequency area; Colombo et al., 2015; Nguyen et al., 2020). In the present study the subjects were lying in a relaxed supine position throughout the recording and were not asked to breathe deeply, they were only asked to change position from supine to sitting for a period of 15 min while eating. The respiration rate was within the range of 9–24 breaths/min almost all of the time. In our study, any incident for which the respiration frequency was out of the range of 9–24 breaths/min was rejected during analysis; hence, there appears to be no benefit in using RFa under our experimental conditions.

### The New Parameters SI/RSA and SI/RMSSD

The two branches of the autonomic nervous system can work reciprocally but they can also work independently. Hence a sympathetic over parasympathetic ratio may not reflect an autonomic “balance” and cannot be used as a singular parameter of autonomic balance. Furthermore, LF and SD2 are not considered good parameters for sympathetic activity, making the LF/HF and SD2/SD1 ratios less useful. These ratios in our study behaved significantly different from SI/RSA and SI/RMSSD. Hence, we calculated SI/RSA and SI/RMSSD *in conjunction with* the SI, RSA and RMSSD values as *additional* parameters to evaluate changes in autonomic activity. The SI/RSA and the SI/RMSSD decreased markedly with the HAPWs confirming a shift to parasympathetic activity.

In conclusion, we show that HAPWs are associated with measurable changes in HRV parameters reflecting parasympathetic and sympathetic activity. Most of the single HAPWs reported here were not noticed by the subjects, they occurred without discomfort or change in respiration pattern. Under normal conditions, they would just be part of everyday movement of content into anal direction without urge to defecate. These motor patterns were not associated with a change in heart rate, suggesting a physiological correlation between the HAPW, the gastrocolic reflex, the sacral defecation reflex, and the autonomic parameters RSA, RMSSD and SI. Our inference is that these motor patterns and reflexes are directed by autonomic activity reflected in HRV, hence RSA, RMSSD, SI, SI/RSA, and SI/RMSSD may develop as biomarkers of autonomic (dys)regulation of colonic motility.

## Data Availability Statement

The raw data supporting the conclusions of this article will be made available by the authors, without undue reservation.

## Ethics Statement

The studies involving human participants were reviewed and approved the by Hamilton Integrated Research Ethics Board. The patients/participants provided their written informed consent to participate in this study.

## Author Contributions

MKA analyzed all the data, incorporated the Baevsky’s Stress Index, and wrote the first draft of the manuscript. LL assisted with data analysis and manuscript writing. J-HC directed and performed all volunteer HRCM studies and discussed manuscript writing. JDH oversaw the autonomic nervous system analysis and manuscript writing. All authors approved the manuscript.

## Conflict of Interest

The authors declare that the research was conducted in the absence of any commercial or financial relationships that could be construed as a potential conflict of interest.

## Abbreviations

HAPW: High-Amplitude Propagating Pressure Wave
HAPW-SPW: High-Amplitude Propagating Pressure Wave followed by a Simultaneous Pressure Wave
HRV: Heart Rate Variability
RSA: Respiratory Sinus Arrythmia
SI: Baevsky’s Stress Index or Sympathetic Index
RMSSD: Root Mean Square of Successive Differences
PEP: Pre-Ejection Period
HF: High Frequency
LF: Low Frequency
SD1: Standard deviation of minor axis of Poincare’ Plot
SD2: Standard deviation of major axis of Poincare’ Plot.

## References

[B1] Baek H. J. Cho C. H. Cho J. Woo J. M. (2015). Reliability of ultra-short-term analysis as a surrogate of standard 5-min analysis of heart rate variability. *Telemed. J. E Health* 21 404–414. 10.1089/tmj.2014.0104 25807067

[B2] Baevsky R. M. Chernikova A. G. (2017). Heart rate variability analysis: physiological foundations and main methods. *Cardiometry* 66–67. 10.12710/cardiometry.2017.10.6676

[B3] Bharucha A. E. Brookes S. J. H. (2018). “Neurophysiologic mechanisms of human large intestinal motility,” in *Physiology of the Gastrointestinal Tract*, ed Said H. (New York, NY: Elsevier), 517–564. 10.1016/b978-0-12-809954-4.00023-2

[B4] Bharucha A. E. Camilleri M. Low P. A. Zinsmeister A. R. (1993). Autonomic dysfunction in gastrointestinal motility disorders. *Gut* 34 397–401. 10.1136/gut.34.3.397 8472990 PMC1374149

[B5] Brookes S. Chen N. Humenick A. Spencer N. J. Costa M. (2016). Extrinsic sensory innervation of the gut: structure and function. *Adv. Exp. Med. Biol.* 891 63–69. 10.1007/978-3-319-27592-5_7 27379635

[B6] Browning K. N. Travagli R. A. (2014). Central nervous system control of gastrointestinal motility and secretion and modulation of gastrointestinal functions. *Compr. Physiol.* 4 1339–1368. 10.1002/cphy.c130055 25428846 PMC4858318

[B7] Browning K. N. Travagli R. A. (2019). Central control of gastrointestinal motility. *Curr. Opin. Endocrinol. Diabetes Obes.* 26 11–16. 30418187 10.1097/MED.0000000000000449 PMC6512320

[B8] Cacioppo J. T. Berntson G. G. Binkley P. F. Quigley K. S. Uchino B. N. Fieldstone A. (1994). Autonomic cardiac control. II. Noninvasive indices and basal response as revealed by autonomic blockades. *Psychophysiology* 31 586–598. 10.1111/j.1469-8986.1994.tb02351.x 7846219

[B9] Callaghan B. Furness J. B. Pustovit R. V. (2018). Neural pathways for colorectal control, relevance to spinal cord injury and treatment: a narrative review. *Spinal Cord* 56 199–205. 10.1038/s41393-017-0026-2 29142293

[B10] Camilleri M. Balm R. K. Low P. A. (1993). Autonomic dysfunction in patients with chronic intestinal pseudo-obstruction. *Clin. Auton. Res.* 3 95–100. 10.1007/bf01818993 8324379

[B11] Chen J.-H. Yu Y. Yang Z. Yu W.-Z. Chen W. L. Kim M. J. M. (2017). Intraluminal pressure patterns in the human colon assessed by high-resolution manometry. *Sci. Rep.* 7:41436. 10.1038/srep41436 28216670 PMC5316981

[B12] Colombo J. Arora R. DePace N. L. Vinik A. I. (2015). *Clinical Autonomic Dysfunction. Measurement, Indications, Therapies, and Outcomes.* Heidelberg: Springer.

[B13] De Groat W. C. Krier J. (1976). An electrophysiological study of the sacral parasympathetic pathway to the colon of the cat. *J. Physiol.* 260 425–445. 10.1113/jphysiol.1976.sp011523 185366 PMC1309099

[B14] De Groat W. C. Krier J. (1978). The sacral parasympathetic reflex pathway regulating colonic motility and defaecation in the cat. *J. Physiol.* 276 481–500. 10.1113/jphysiol.1978.sp012248 650474 PMC1282439

[B15] Devroede G. Giese C. Wexner S. D. Mellgren A. Coller J. A. Madoff R. D. (2012). Quality of life is markedly improved in patients with fecal incontinence after sacral nerve stimulation. *Female Pelvic Med. Reconstr. Surg.* 18 103–112. 10.1097/spv.0b013e3182486e60 22453321

[B16] Devroede G. Lamarche J. (1974). Functional importance of extrinsic parasympathetic innervation to the distal colon and rectum in man. *Gastroenterology* 66 273–280. 10.1016/s0016-5085(74)80114-9 4810918

[B17] Dinning P. G. Sia T. C. Kumar R. Mohd Rosli R. Kyloh M. Wattchow D. A. (2016). High-resolution colonic motility recordings in vivo compared with ex vivo recordings after colectomy, in patients with slow transit constipation. *Neurogastroenterol. Motil.* 28 1824–1835. 10.1111/nmo.12884 27282132

[B18] Furness J. B. Callaghan B. P. Rivera L. R. (2014). The enteric nervous system and gastrointestinal innervation: integrated local and central control. *Adv. Exp. Med. Biol.* 817 39–71. 10.1007/978-1-4939-0897-4_3 24997029

[B19] Gasparini S. Howland J. M. Thatcher A. J. Geerling J. C. (2020). Central afferents to the nucleus of the solitary tract in rats and mice. *J. Comp. Neurol.* 528 2708–2728. 32307700 10.1002/cne.24927 PMC7942812

[B20] Goldstein D. S. Bentho O. Park M. Sharabi Y. (2011). Low−frequency power of heart rate variability is not a measure of cardiac sympathetic tone but may be a measure of modulation of cardiac autonomic outflows by baroreflexes. *Exp. Physiol.* 96 1255–1261. 10.1113/expphysiol.2010.056259 21890520 PMC3224799

[B21] Grossman P. Taylor E. W. (2007). Toward understanding respiratory sinus arrhythmia: relations to cardiac vagal tone, evolution and biobehavioral functions. *Biol. Psychol.* 74 263–285. 10.1016/j.biopsycho.2005.11.014 17081672

[B22] Hayano J. Yuda E. (2019). Pitfalls of assessment of autonomic function by heart rate variability. *J. Physiol. Anthropol.* 38:3. 10.1186/s40101-019-0193-2 PMC6416928 30867063

[B23] Houtveen J. H. Groot P. F. Geus E. J. (2005). Effects of variation in posture and respiration on RSA and pre-ejection period. *Psychophysiology* 42 713–719. 10.1111/j.1469-8986.2005.00363.x 16364066

[B24] Jean A. (1991). [The nucleus tractus solitarius: neuroanatomic, neurochemical and functional aspects]. *Arch. Int. Physiol. Biochim. Biophys.* 99 A3–A52. 1720691 10.3109/13813459109145916

[B25] Kubios (2020). Available online at: https://www.kubios.com/about-hrv (accessed August 28, 2020).

[B26] La Rovere M. T. Pinna G. D. Maestri R. Mortara A. Capomolla S. Febo O. (2003). Short-term heart rate variability strongly predicts sudden cardiac death in chronic heart failure patients. *Circulation* 107 565–570. 10.1161/01.cir.0000047275.25795.17 12566367

[B27] Leblanc D. McFadden N. Lebel M. Devroede G. (2015). Fecal continence can be restored by sacral neurostimulation after traumatic unilateral pudendal neuropathy: a case report. *Int. J. Colorectal Dis.* 30 569–570. 10.1007/s00384-014-2019-3 25296707

[B28] Lorena S. L. Figueiredo M. J. Almeida J. R. Mesquita M. A. (2002). Autonomic function in patients with functional dyspepsia assessed by 24-hour heart rate variability. *Dig. Dis. Sci.* 47 27–31. 11837729 10.1023/a:1013246900041

[B29] Lu C. L. Zou X. Orr W. C. Chen J. D. (1999). Postprandial changes of sympathovagal balance measured by heart rate variability. *Dig. Dis. Sci.* 44 857–861. 10219849 10.1023/a:1026698800742

[B30] Milkova N. Parsons S. P. Ratcliffe E. Huizinga J. D. Chen J.-H. (2020). On the nature of high-amplitude propagating pressure waves in the human colon. *Am. J. Physiol. Gastrointest. Liver Physiol.* 318 G646–G660. 10.1152/ajpgi.00386.2019 32068445 PMC7191456

[B31] Nguyen L. Wilson L. A. Miriel L. Pasricha P. J. Kuo B. Hasler W. L. (2020). Autonomic function in gastroparesis and chronic unexplained nausea and vomiting: relationship with etiology, gastric emptying, and symptom severity. *Neurogastroenterol. Motil.* 32:e13810. 10.1111/nmo.13810 PMC7377964 32061038

[B32] Ouyang X. Li S. Zhou J. Chen J. D. (2020). Electroacupuncture ameliorates gastric hypersensitivity via adrenergic pathway in a rat model of functional dyspepsia. *Neuromodulation* 23 1137–1143. 10.1111/ner.13154 32282996

[B33] Parsons S. (2019) Available online at: http://scepticalphysiologist.com.html (accessed August 28, 2020).

[B34] Rahman F. Pechnik S. Gross D. Sewell L. Goldstein D. S. (2011). Low frequency power of heart rate variability reflects baroreflex function, not cardiac sympathetic innervation. *Clin. Auton. Res.* 21 133–141. 10.1007/s10286-010-0098-y 21279414 PMC3094491

[B35] Rahman S. Habel M. Contrada R. J. (2018). Poincaré plot indices as measures of sympathetic cardiac regulation: responses to psychological stress and associations with pre-ejection period. *Int. J. Psychophysiol.* 133 79–90. 10.1016/j.ijpsycho.2018.08.005 30107195

[B36] Schächinger H. Weinbacher M. Kiss A. Ritz R. Langewitz W. (2001). Cardiovascular indices of peripheral and central sympathetic activation. *Psychosom. Med.* 63 788–796. 10.1097/00006842-200109000-00012 11573027

[B37] Semba T. Fujii Y. (1970). Relationship between venous flow and colonic peristalsis. *Jpn. J. Physiol.* 20 408–416. 10.2170/jjphysiol.20.408 5312068

[B38] Shaffer F. Ginsberg J. P. (2017). An overview of heart rate variability metrics and norms. *Front. Public Health* 5:258. 10.3389/fpubh.2017.00258 29034226 PMC5624990

[B39] Shimizu Y. Chang E. C. Shafton A. D. Ferens D. M. Sanger G. J. Witherington J. (2006). Evidence that stimulation of ghrelin receptors in the spinal cord initiates propulsive activity in the colon of the rat. *J. Physiol.* 576 329–338. 10.1113/jphysiol.2006.116160 16873401 PMC1995628

[B40] Singh A. Jaryal A. K. (2020). “Neurophysiology of Respiratory System,” in *Brain and Lung Crosstalk*, eds Prabhakar H. Mahajan C. (Singapore: Springer), 1–38. 10.1007/978-981-15-2345-8_1

[B41] Smith-Edwards K. M. Najjar S. A. Edwards B. S. Howard M. J. Albers K. M. Davis B. M. (2019). Extrinsic primary afferent neurons link visceral pain to colon motility through a spinal reflex in mice. *Gastroenterology* 157 522–536.e2. 31075226 10.1053/j.gastro.2019.04.034 PMC6995031

[B42] Szurszewski J. Miller S. M. (2006). “Physiology of prevertebral sympathetic ganglia,” in *Physiology of the Gastrointestinal Tract*, ed. Johnson L. R. (San Diego, CA: Academic Press), 603–627. 10.1016/b978-012088394-3/50025-8

[B43] Taché Y. Million M. (2015). Role of corticotropin-releasing factor signaling in stress-related alterations of colonic motility and hyperalgesia. *J. Neurogastroenterol. Motil.* 21 8–24. 25611064 10.5056/jnm14162 PMC4288101

[B44] Thayer J. F. Ahs F. Fredrikson M. Sollers J. J. Wager T. D. (2012). A meta-analysis of heart rate variability and neuroimaging studies: implications for heart rate variability as a marker of stress and health. *Neurosci. Biobehav. Rev.* 36 747–756. 10.1016/j.neubiorev.2011.11.009 22178086

[B45] Van Lien R. Neijts M. Willemsen G. de Geus E. J. (2015). Ambulatory measurement of the ECG T-wave amplitude. *Psychophysiology* 52 225–237. 10.1111/psyp.12300 25123155

[B46] Yuan Y. Ali M. K. Mathewson K. J. Sharma K. Faiyaz M. Tan W. (2020). Associations between colonic motor patterns and autonomic nervous system activity assessed by high-resolution manometry and concurrent heart rate variability. *Front. Neurosci.* 13:1447. 10.3389/fnins.2019.01447 32038145 PMC6989554

